# Current Perspectives on Percutaneous Vertebroplasty: Current Evidence/Controversies, Patient Selection and Assessment, and Technique and Complications

**DOI:** 10.1155/2011/175079

**Published:** 2011-05-21

**Authors:** Robert James Nairn, Shagran Binkhamis, Adnan Sheikh

**Affiliations:** Musculoskeletal Division, Department of Diagnostic Imaging, The Ottawa Hospital, General Campus, 501 Smyth Road, Ottawa, ON, Canada K1H 8L6

## Abstract

Osteoporotic-associated vertebral compression fractures are a major public health concern, dwarfing even hip fractures in incidence in the United States. These fractures carry a significant morbidity and mortality burden and also represent a major growing source of consumption of scarce heath resources. Percutaneous vertebroplasty remains a commonly used and safe technique for the symptomatic treatment of vertebral compression fractures, both osteoporotic- and neoplastic-induced. By carefully selecting appropriate patients who are referred promptly, vertebroplasty can provide significant and durable pain relief over traditional conservative therapy. Recent controversies surrounding the evidence for vertebroplasty in osteoporotic-associated vertebral compression fractures are reviewed. A comprehensive step-by-step practical guide to performing vertebroplasty is then described. A brief description of patient selection, workup, as well as complications is also provided.

## 1. Introduction

Two related interventional procedures are performed for the treatment of painful vertebral compression fractures (VCF), percutaneous vertebroplasty (PV), and kyphoplasty. Both involve the fluoroscopically or CT-guided percutaneous placement of wide-bored cannulae into the fractured vertebral body which is subsequently cemented with synthetic bone cement, typically polymethylmethacrylate (PMMA). PV was first described for the treatment of an aggressive haemangioma of C2 in 1987 [1, 2], but in subsequent years has been performed mainly for the symptomatic treatment of painful osteoporotic-induced VCF. The palliative treatment of tumour-induced VCF (typically myeloma or metastatic disease) remains an important further indication for PV.

VCF is a major public health concern, with an estimated 1.4 million patients presenting with such a fracture each year [3]. The incidence of VCF in the US alone is estimated to be 750,000 [4], a figure that makes VCF more common than osteoporotic-related hip fractures. The majority of these vertebral fractures are secondary to osteoporosis. Gradual pain resolution following these fractures is the expected natural history, but pain can persist and or resolve slowly [5]. Pain in these patients is also frequently refractory to conservative therapy. VCFs can be complicated by deformity, loss of stature, impairment of pulmonary function and the attendant risks of poor mobility/immobilisation in the elderly, such as venous thrombo-embolism [6–8]. Immobilisation is also associated with increased bone density loss and enduring difficulty with activities of daily living [9]. An increase in mortality after VCF has been noted. One large prospective cohort study of elderly patients with low-trauma osteoporotic VCFs calculated an increased standardized mortality ratio for women of 1.82 (95% CI 1.52–2.17) and for men of 2.12 (95% CI 1.66–2.72) [10].

Traditional treatment of uncomplicated VCF consists of analgesia, bracing, and rehabilitation. Pain often necessitates bed rest or other restrictions on mobility. Open reduction and internal fixation is theoretically possible, but rarely performed due to poor bone stock and multiple underlying co-morbidities. PV offers a less invasive therapeutic option for generally elderly patients with multiple comorbidities which allows for early immobilisation.

## 2. Evidence and Controversies

Prior to 2009, evidence for the effectiveness of PV in osteoporotic VCF was based on anecdotal experience, multiple prospective and retrospective case series, and prospective comparative cohort studies [11]. Multiple observational studies had shown almost uniformly excellent results with PV, with moderated to marked pain relief experienced by 75–95% of patients [12–14]. Similar results in the treatment of metastatic fractures have also been reported [15]. However, a bias towards overestimation of treatment benefits is possible when relying on this form of evidence [16, 17]. Well-designed, randomised, prospective trials comparing PV versus conservative therapy were lacking. In 2009, NEJM published the results of the first two randomised blinded trials comparing PV with a sham intervention, namely, local anaesthetic infiltration of skin, subcutaneous and periosteum [18, 19].

Buchbinder et al. [18] enrolled and randomised 78 patients with one or two painful osteoporotic vertebral fractures (as defined on imaging, predominately MRI) to either PV or a sham procedure, with followup reported up to six months. Kallmes et al. [19] enrolled and randomised 131 patients with one to three fractures to PV or a similar sham procedure; followup was to three months. Both trials reported no statistically significant benefit of PV over placebo.

The NEJM trials have been criticised on several fronts, with Buchbinder in particular strongly defending the validity of the trials in ensuing correspondence [20, 21]. Kallmes et al. [19] allowed cross-over between the two arms at one month. For those receiving the sham procedure, 42% opted to receive PV at three months, compared with 12% for the other arm. This higher rate of cross-over could be interpreted as reflecting dissatisfaction with the sham procedure compared with PV, or possibly flaws in the blinding of the sham procedure such that patients were able to “guess” their intervention.

Mathis [22] and Munk et al. [23] criticised the low patient enrolment number compared with length of the enrolment period. Conceivably, patients with the most severe pain would be less likely to agree to undergo randomisation, and the studied population would therefore exclude those patients with the most severe pain. These authors also point out that Kallmes was close to proving a “clinically meaningful improvement in pain for PV compared to sham therapy” with a *P* value of  .06. If the Kallmes trial had enrolled 19 more participants who experienced similar results, *P* would have been <.05 and thus regarded as statistically significant. Mathis concluded that Kallmes therefore did not enrol enough patients to disprove the effectiveness of PV. The number of patients enrolled in Buchbinder was also insufficient to power a subgroup analysis to assess PV effectiveness in those with fractures less than six weeks old [24]. The NEJM trials have also been criticised on the basis of the proportion of eligible patients who declined enrolment, 70% for Kallmes and 64% for Buchbinder. Bono et al. [25] suggested this raised the spectre of selection bias. However, these figures are not remarkable when compared with previous experience in blinded randomised controlled trials.

The expected natural history of VCF is of gradual healing and pain relief [26, 27]. Some evidence from observational studies of PV have shown decreased pain relief and less improvement in mobility in fractures >12 months when treated with PV [28]. Based on the patient selection criteria of both NEJM studies, patients with painful fractures up to one year of age were included in the trials. Most patients had pain of >3 months duration. This would have inevitably resulted in the enrolment of patients with delayed union and non union, altering the homogeneity of the disease process studied. The average reduction in pain as assessed on a visual analogue scale (VAS) was smaller in the active arms of both trials than would have been expected from prior observational studies, raising doubt about the ability to extrapolate these results to patients with severe pain post-VCF who have failed conservative treatment. Bono et al. also criticised the design of the sham procedure, labelling it an alternative intervention (the injection of long acting local anaesthetic onto periosteum) rather than a true sham procedure. It is difficult to imagine, however, how participants in the NEJM could have been truly blinded without recourse to such a procedure. Some authors, including the current authors, advocate fluoroscopic-guided palpation and percussion of PV candidates, in an attempt to correlate imaging findings with symptoms. This was not performed in either study. Both trials also did not examine the role of PV in nonosteoporotic vertebral fractures, or examine the role of PV in the inpatient setting.

More recently, the results of VERTOS II [29] have become available. This multicenter study was randomised, but participants, physicians and outcome assessors were not blinded. Over 200 patients with a VCF and pain of less than 6 weeks duration were randomised to conservative treatment or PV. No sham procedure was performed. At one month and one year, a statistically significant reduction in pain in the PV arm was evident. Alvarez et al. [11] concluded that PV is safe and able to be performed at an acceptable cost. Furthermore the pain relief following PV is immediate, lasts for at least a year, and is significantly greater than conservative treatment. 

Rousing et al. [30] also recently reported 12-month followup from an open-label, randomised study (*n* = 50, fracture age <8 weeks) comparing PV with conservative management. Immediate and significant pain relief following PV was evident. At one-month following discharge, the PV arm had a statistically significantly reduction in pain compared with the conservative therapy arm. However, no difference in pain scores was found between the groups at 3 and 12 months. These authors suggest that the role of PV may therefore be as a short-term invasive method of pain control in those who fail conservative treatment or for those whom conservative treatment and the accompanying immobilisation carry serious risks.

## 3. Conclusions on Available Evidence for PV

**Figure 1:**
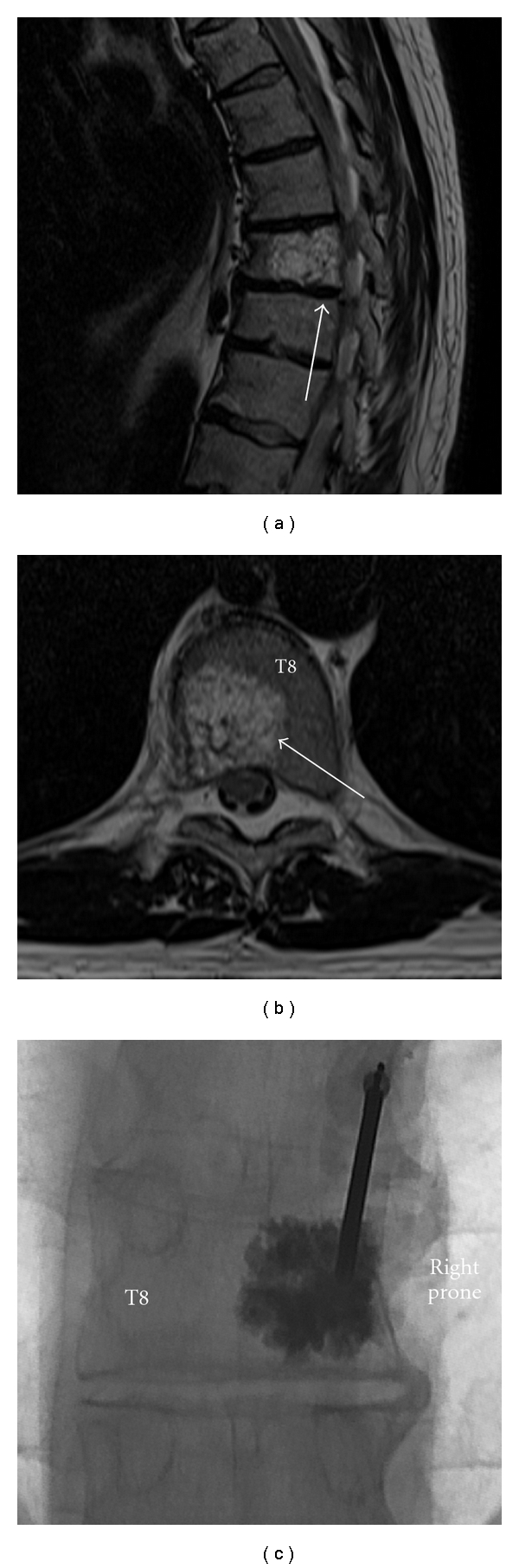
Painful haemangioma of right-hand aspect of T8 (arrowed) in a 56-year-old man treated with unipedicular vertebroplasty. Sagittal (a) and axial (b) TSE T2-weighted images. (c): Vertebroplasty spot fluoroscopy image, AP projection. Postprocedure, the patient reported complete resolution of pain.

It can be concluded from the available literature that long-term effectiveness and complication data from PV is currently lacking. Performing a true blinded randomised-controlled trial between conservative therapy and PV is impossible. Despite the seemingly conflicting available data, it is the authors' opinion that there is some evidence available that in the acute to subacute period, in those who are failing conservative treatment or at are at increased risk from immobilisation, PV can provide good pain relief compared with conservative treatment, though as yet no durable long-term benefit has been demonstrated. When patients are carefully screened by history, examination, and imaging prior to the procedure, it is the authors' opinions that a group highly likely to significantly benefit from PV can routinely be identified and then offered PV. PV remains an important intervention for the treatment of intractable pain associated with neoplasm-induced VCF (Figure 1), and for those hospitalised due to severe pain following osteoporotic-induced VCF.

## 4. Performing Percutaneous Vertebroplasty

### 4.1. Patient Selection

Ideally, patients with painful VCF who are failing conservative treatment should be referred for PV as early as practicable once it has become clear that conservative measures are failing, or in cases of severe pain requiring hospitalisation. Patients with pain of greater than three months duration are less likely to benefit from PV [29].

There are few absolute contraindications to PV. Coagulation profile must be normal or near normal, and anticoagulants must be ceased prior to the procedure. PV is contraindicated in those with severe cardiac or respiratory failure precluding safe conscious sedation or general anaesthesia. These patients are considered high risk for clinically significant procedure-related fat embolism [31]. Infection or fever is also an absolute contraindication. Severe loss of vertebral body height is a relative contraindication, but good results can often be obtained in even severely compressed vertebrae [32].

**Figure 2:**
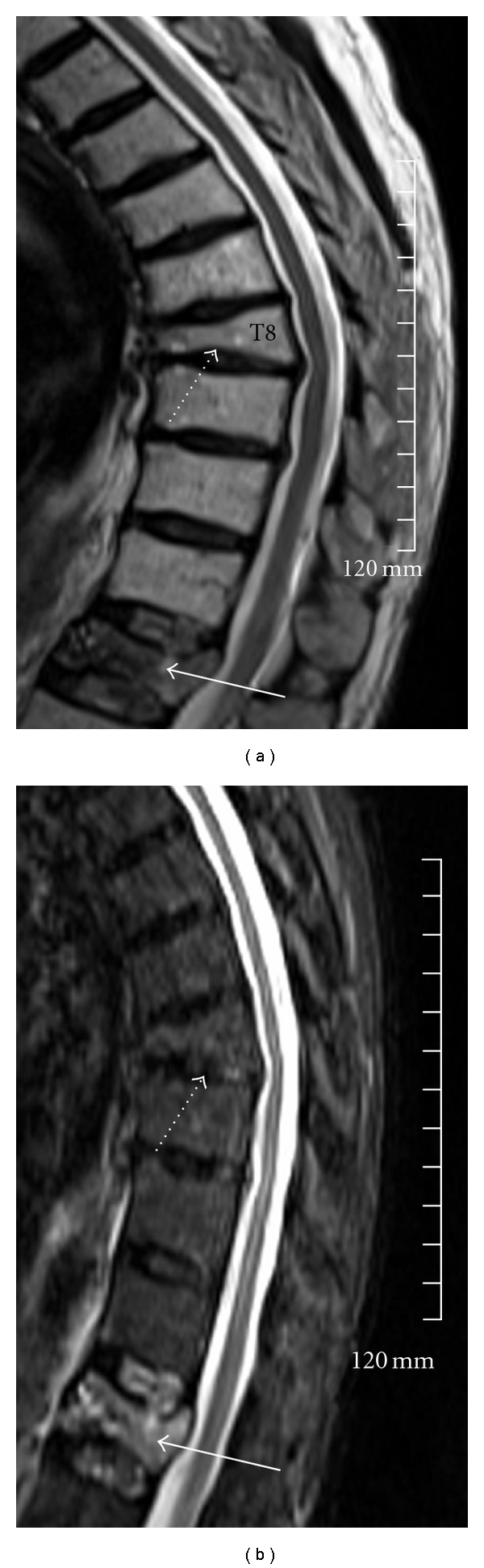
Sagittal TSE T2-weighted (a) and sagittal STIR (b) of the thoracolumbar spine in a 56-year-old man referred for PV for severe back pain following a fall. VCFs are noted at T8 (white dashed arrow) and T12 (continuous white arrow). There is prominent STIR hyperintensity at T12 without significant marrow edema at T8. The patient was symptomatic over T12 on palpation. This level was cemented with complete resolution of symptoms.

Formal prevertebroplasty assessment is performed by the interventionist one to two weeks before the procedure is scheduled. At this time, all of the imaging is reviewed. It is vital to ensure that a recent MRI dating from the time of symptom onset is available to assess marrow edema in the target levels. PV is contraindicated if bone marrow edema is absent on STIR (short-tau inversion recovery) images (Figure 2). If MRI is contraindicated or unavailable, imaging assessment can be performed with a combination of CT and scintigraphic bone scan. CT may also be useful for assessing pedicle bone stock and the integrity of the posterior vertebral body cortex in selected patients, particularly in patients with malignant VCF. Severe loss of bone stock of the pedicle and posterior vertebral body cortex is a relative contraindication to PV due to increased risk of symptomatic cement leak.

A comprehensive history is obtained and a thorough examination is performed. The examination is performed, in part, in the fluoroscopic suite with the patient prone. Useful clinical information can be obtained prior to this stage by observing the pain induced by patient mobilising and climbing onto the fluoroscopy table. It is important to careful correlate the region of marrow edema on cross-sectional imaging with the fluoroscopic image of the vertebra. The patient is then palpated and or gently percussed in the region of fracture in an attempt to correlate symptoms with imaging findings. If there is a clear disparity between the imaging and the examination findings, PV is contraindicated. Signs and symptoms of concurrent facet or sacroiliac joint dysfunction, radiculopathy, or sacral insufficiency fracturing should be considered a relative contraindication to PV and prompt careful reassessment of the patient's symptoms.

An assessment of the likely fluoroscopic approach to PV is made to ensure the procedure is technically feasible. Severe kyphosis and scoliosis are a relative contraindication. Poor pedicle visualisation and or aberrant anatomy may require CT-guided placement of bone cannulae. If the patient is unable to lie prone, general anaesthesia may be indicated. If the procedure is technically feasible and the imaging findings correlate with the examination findings, the patient is then educated about the procedure and possible complications, and informed consent is obtained.

### 4.2. Procedure

PV entails the percutaneous injection of bone cement, typically PMMA, into the collapsed vertebral body. In our institution, PV is generally performed under general anaesthesia, although the procedure can be safely performed under conscious sedation. After prone positioning, the X-ray tube and image-intensifier is centered over the relevant vertebral body such that the superior and inferior endplates are aligned/not obliqued and the spinous process is midline. The pedicle to be cannulated is then centred in the vertebral body image via cephalo-caudal tube angulation. Right and left anterior oblique tube positioning is performed to visualise the relevant pedicle “en-face.” If biplane is available, the true lateral projection of the vertebral body is obtained by ensuring that the posterior vertebral cortex is visualised in profile without obliquity, the left and right ribs at the target level (if visible) completely overlap each other and the target vertebral body is centered in the image. PV can be safely performed without access to biplane fluoroscopy but is significantly more time consuming. 

**Figure 3:**
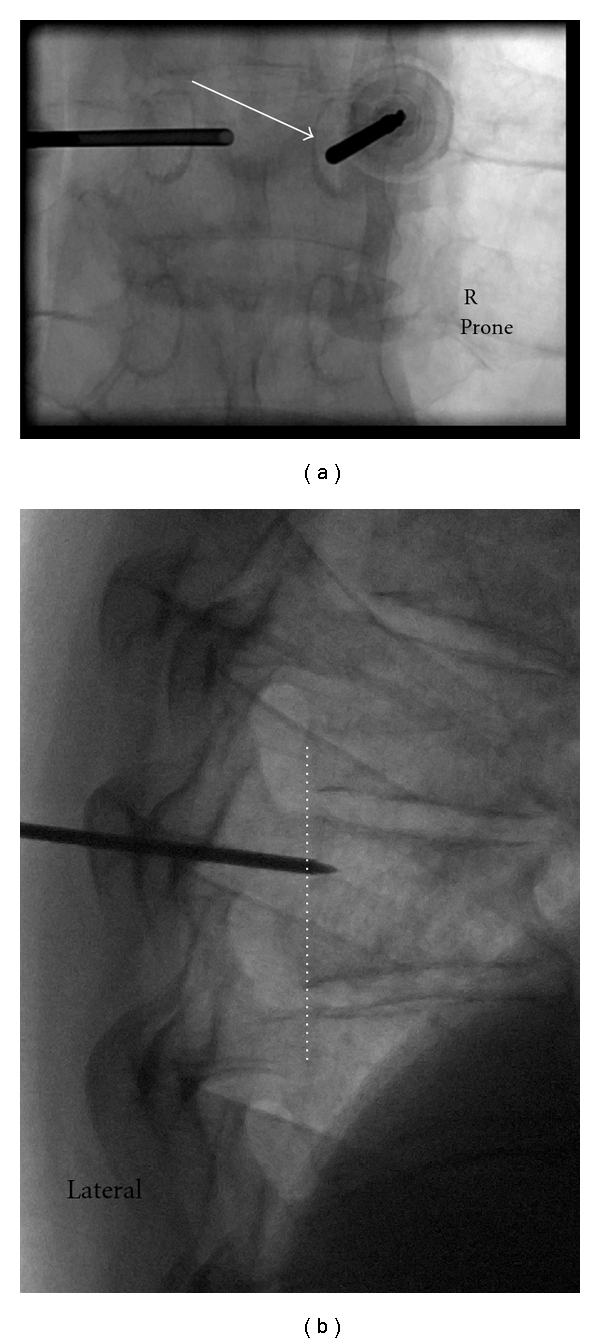
Spot fluoroscopic images from two separate patients obtained during PV. (a) demonstrates left bone cannula insitu. The right pedicle has been cannulated, and is advanced in the AP plane staying clear of the pedicle medial cortical margin. Arrow indicates the medial pedicle cortex. (b) Lateral projection demonstrates tip of needle anterior to posterior vertebral cortex (dashed white line). Once this landmark is reached, the cannula can be safely advanced to 1 cm posterior to the anterior vertebral cortex under lateral projection fluoroscopic guidance. Cementing is also performed in this projection.

With aseptic technique and following local anaesthesia, an 11 G or 13 G bone needle is inserted down onto the periosteum overlying the pedicle, aiming for the lateral cortical margin of the pedicle at the 10:00 position on the left and the 2:00 position on the right. A transpedicular approach is the technique of choice. This approach ensures that risk of damage to adjacent structures is minimised, is easily learnt, and conveniently positions the cannula tip at maximal distance from bone entry for cement injection, helping to minimise leaks. Occasionally, a parapedicular approach may be necessary due to difficult/distorted anatomy. Cannula advancement is then performed. Typically, the periosteum is breached and the cannula advanced with hand pressure and screwing. Soft osteoporotic bone means that a soft bone mallet is only occasionally needed for cannula advancement. An attempt to stay initially lateral to the midline of the pedicle on the oblique AP projection is made to minimise the chances of breaching the medial cortex of the pedicle, and thus entering the spinal canal, during cannula positioning (Figure 3(a)). After complete cannula engagement of bone, the true lateral projection is checked for cannula trajectory in the cephalo-caudal plane. Once the needle is anterior to the posterior margin of the vertebral body on the lateral projection, the pedicle has been successfully traversed and further needle progression can be monitored via the lateral projection (Figure 3(b)). Prior to this landmark, cannula progression should only be performed in the oblique AP projection, staying clear of the medial pedicle cortical margin.

The tip of the cannula is advanced to a point approximately 1 cm posterior to the anterior vertebral body as assessed laterally. Because the vertebral body is not rectangular but rather curved, leaving a safe gap between these two points is needed to ensure the anterior cortex is not breached. The contralateral pedicle is cannulated in a similar fashion. Bilateral cannulation is favoured due to increased chance of adequate and safe cement injection. If there is doubt about the diagnosis, bone biopsy is performed at this stage. 

The PMMA cement is prepared by mixing polymer powder with liquid monomer. Mixing of the powder and liquid leads to exothermic polymerisation and then progressively thickening of the paste, which subsequently hardens. Once the cement has a toothpaste-like consistency, injections commence via a 1 mL syringe. Continuous screening for cement extravasation during injection is performed. In particular, close attention is made to the posterior margin of the vertebral body. If cement extends posteriorly within 5 mm of this landmark, injection is ceased to minimise the risk of epidural space cement leak. One needle only at a time is injected. Any leak or extravasation is an indication to stop injection. Generally, a small waiting period for the cement to solidify is all that is required prior to resuming injection. Large leaks may require the procedure be abandoned. At the end of the procedure when the cannulae are removed, it is advisable to reinsert the introducing stylet to push any residual cement into the needle tract within the bone. A cast of cement deposited into soft tissues is irritant and can cause significant pain. 

### 4.3. Volume of PMMA to Inject

**Figure 4:**
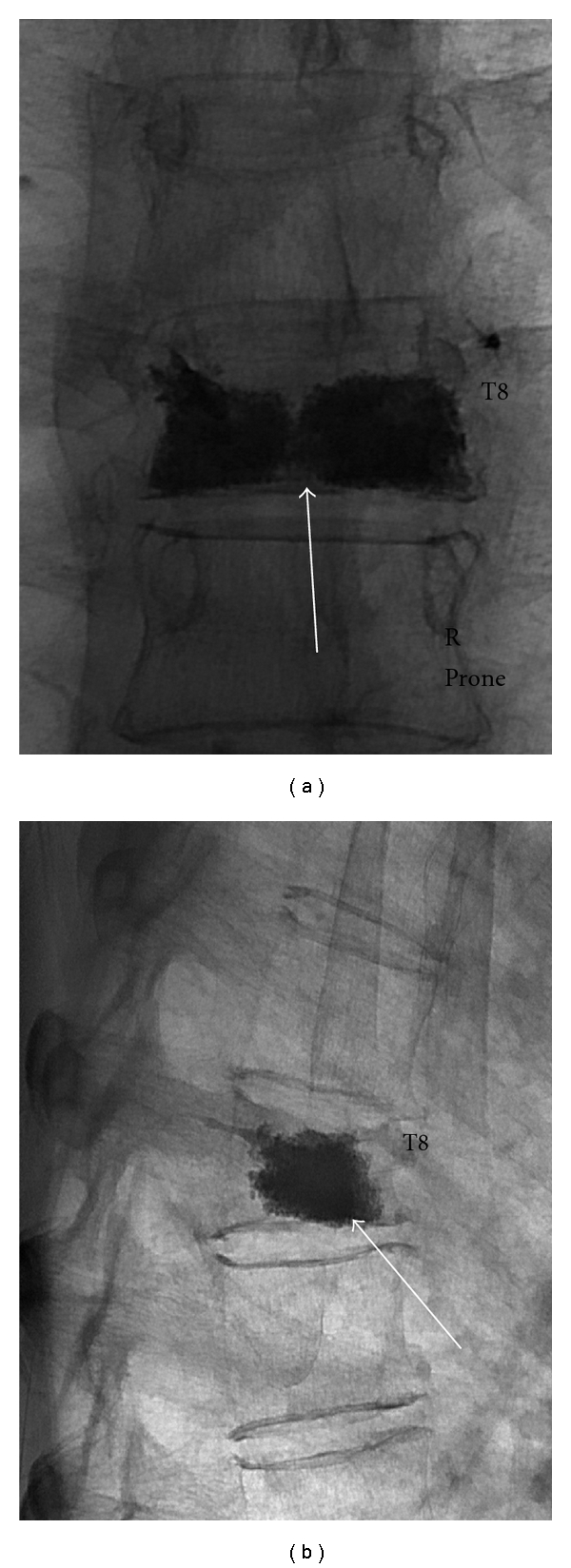
Spot AP (a) and lateral (b) fluoroscopic images following T8 PV in a 62-year-old man with an osteoporotic VCF. A bipedicular approach was used. There is satisfactory filling of the vertebral body in the lateral and AP projections without evident leak (arrows). The patient's symptoms had resolved on awakening from the procedure.

The volume of cement to be injected at each level for maximal efficacy has not been accurately determined [33]. In ex vivo studies, Belkoff et al. [33] demonstrated that vertebral body strength can be restored with 2 mL of cement, whilst restoration of preinjury stiffness required 4–8 mL. Regardless of exact measurements, visual filling of the body on the AP projection from the inner margins of the endplates along the lateral 1/3 on each side is ideal, though this is not always technically feasible (Figure 4) [31]. The current authors have often found this result to be achievable with 3–5 mL of cement. As pain relief does not correlate with volume of injected cement [12], visual inspection of the amount of cement injected to the residual volume of the compressed vertebra is recommended when ascertaining total volume of cement to be injected.

### 4.4. Postprocedure Care

The patient remains supine or semirecumbent for one hour, with monitoring of neurovascular status and wound inspection every 15 and 30 minutes respectively. The patient is then gently mobilised. If the status of the patient is stable, they can be discharged home after two hours. Occasionally, patients may experience an increase in pain following PV. This is usually of benign aetiology and self-limiting [22]. Good pain relief is usually obtained with oral or parenteral narcotics. Ongoing severe pain, radicular pain or signs/symptoms of spinal stenosis should prompt early imaging with CT. Followup for treatment of the patient's underlying osteoporosis is mandatory.

### 4.5. Complications

In general, PV is a safe procedure which is well tolerated. The overall complication rate in PV for VCF reported in the literature is low, ranging from 1% to 10% [13, 34–36]. Complications are considerably more common in the acute peri-and postoperative period. The complication rate for malignant tumours is higher than for benign VCF [36–41], with one review by Murphy and Deramond [41] estimating the complication rate for OP to be 1.3% for osteoporotic VCFs, 2.5% for haemangiomata-associated VCF, and 10% for malignant VCF. 

**Figure 5:**
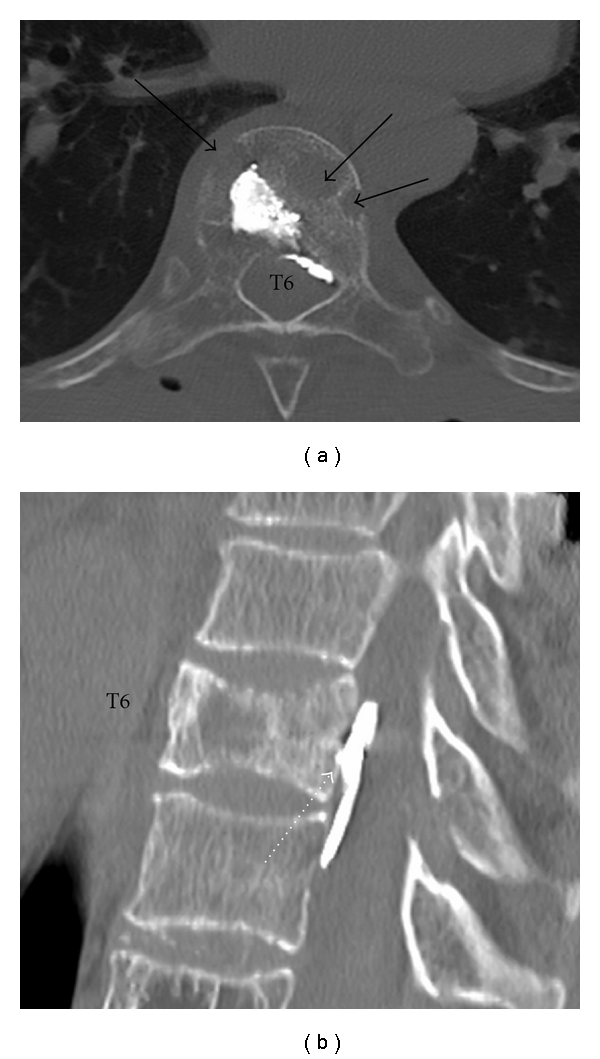
86-year-old woman with known multiple myeloma and pathological fracture of T6. During PV, posterior cement leak was noted soon after injection commenced; the procedure was abandoned. (a): Axial CT demonstrates permeative bone destruction of T6 (black arrows). Cement has extravasated posteriorly into the epidural space. (b) Sagittal CT demonstrates a thin collection of epidural cement anterior to the thecal sac (dashed white arrow). The patient remained asymptomatic initially and at followup and decompression was not required.

**Figure 6:**
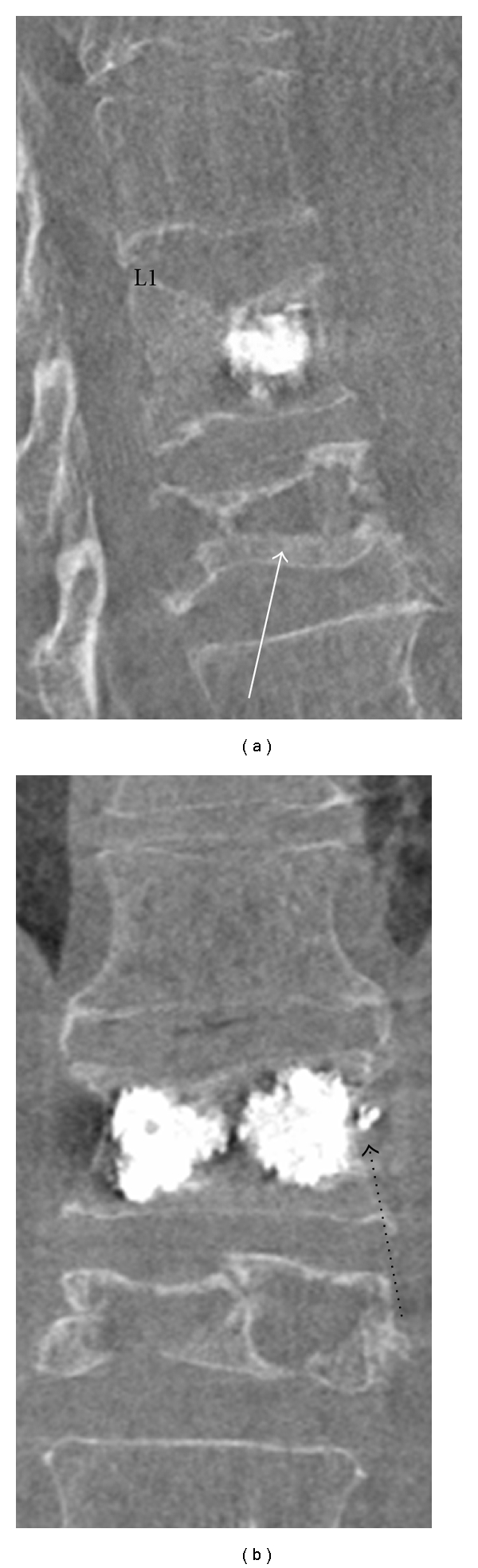
Modern biplane fluoroscopy allows on-table CT to be performed, with multiplanar reformats. Although the images acquired are susceptible to quantum mottle, the technique is invaluable in PV to quickly assess and confirm cement position and to assess for complications. Sagittal (a) and coronal (b) reconstructions (Siemens Axiom Artis biplane fluoroscopic unit) are shown in a 78-year-old man with recent VCF of L1 treated successfully with PV. An old VCF is noted at L2 (white arrow). A trace of lateral cement leak is noted to the left (dashed black arrow). This was asymptomatic.

The commonest complication of PV is cement extravasation, occurring 26–97% of the time [42]. The vast majority of leaks are asymptomatic. However, cement leaks can narrow and/or impinge neural structures, either within the neural foramina or the epidural space (Figures 5 and 6). [43, 44]. Radiculopathy related to cement leakage is usually transient and responds well to systemic analgesia and or transforaminal nerve block, although occasional cases requiring of surgical decompression have been reported [43, 44]. Failure to respond promptly to transforaminal nerve block for radiculopathy or any symptoms referable to central canal stenosis is an indication for urgent neurosurgical consultation and decompression.

Systemic cement embolisation is rare, with one study estimated as much as 5% of patients may undergo cement pulmonary embolism [45]. These are rarely clinically significant [46]. Displaced marrow from the cemented vertebra may also result in fat embolism. Patients with poor cardiorespiratory function may be at increased risk of this complication [46]. As risk of marrow embolisation may be proportional to volume of displaced marrow during cement injection, the authors favour performing PV at up to three levels only at one sitting. Venography prior to cementing has fallen out of favour [47, 48] as no clinical benefit referable to venography has been demonstrated [28]. Other complications of PV include bleeding, haematoma, infection, pneumothorax, pedicle fracture, thecal sac puncture, and CSF leak. Patients with severely lowered bone mineral density may experience fractured ribs or sternum whilst the procedure is performed [49]. Infection is uncommon, and there is no evidence to support routine antibiotic impregnation into the cement or intravenous antibiotics at the time of the procedure [35].

PMMA used during arthroplasty has been associated with transient hypotension, though a link between PV with PMMA and cardiovascular effects has not been demonstrated on retrospective review [50]. The exothermic process of PMMA polymerisation has led to some concern about possible associated thermal injury, but in experimental testing no thermal injury to neurological structures has been demonstrated [51, 52].

### 4.6. Risk of Subsequent Fracture at Adjacent Vertebrae

Some reports in the literature have suggested that PV is associated with a higher incidence of new VCF at adjacent levels, possibly reflecting a biomechanical consequence of the augmented stiffness of the cemented level [53–56]. Recent data from the VERTOS II trial [57] randomising painful VCFs to conservative treatment or PV showed the incidence of new VCF between the two groups was not different at 12 months followup, and the only risk factor identified for new VCF was the number of baseline VCFs. This adds weight to the theory that new VCF post-PV are manifestations of the natural history of osteoporosis rather than reflecting a risk intrinsic to PV.

## 5. Conclusion

Prompt assessment of PV candidates is integral to successfully performing the procedure. The interventionist must take responsibility for reviewing imaging, taking a thorough history and examining the patient. Percutaneous vertebroplasty is usually technically straight forward and safe as long as a clear, reproducible, step-by-step approach is employed. Serious complications are uncommon and pain relief induced is often considerable. 

## References

[1] Galibert P, Deramond H, Rosat P, Le Gars D (1987). Preliminary note on the treatment of vertebral angioma by percutaneous acrylic vertebroplasty. *Neurochirurgie*.

[2] Cortet B, Cotten A, Deprez X (1994). Value of vertebroplasty combined with surgical decompression in the treatment of aggressive spinal angioma. Apropos of 3 cases. *Revue du Rhumatisme*.

[3] Johnell O, Kanis JA (2006). An estimate of the worldwide prevalence and disability associated with osteoporotic fractures. *Osteoporosis International*.

[4] Melton LJ, Thamer M, Ray NF (1997). Fractures attributable to osteoporosis: report from the national osteoporosis foundation. *Journal of Bone and Mineral Research*.

[5] Silverman SL, Minshall ME, Shen W, Harper KD, Kie S (2001). On behalf of the health-related quality of life subgroup of the Multiple Outcomes of Raloxifene Evaluation Study. The relationship of health-related quality of life to prevalent and incident vertebral fractures in postmenopausal women with osteoporosis: results form the Multiple Outcomes of Raloxifene Evaluation Study. *Arthritis & Rheumatism*.

[6] Lyritis GP, Mayasis B, Tsakalakos N (1989). The natural history of the osteoporotic vertebral fracture. *Clinical Rheumatology*.

[7] Schlaich C, Minne HW, Bruckner T (1998). Reduced pulmonary function in patients with spinal osteoporotic fractures. *Osteoporosis International*.

[8] Lyles KW, Gold DT, Shipp KM, Pieper CF, Martinez S, Mulhausen PL (1993). Association of osteoporotic vertebral compression fractures with impaired functional status. *American Journal of Medicine*.

[9] Silverman SL (1992). The clinical consequences of vertebral compression fracture. *Bone*.

[10] Bliuc D, Nguyen ND, Milch VE, Nguyen TV, Eisman JA, Center JR (2009). Mortality risk associated with low-trauma osteoporotic fracture and subsequent fracture in men and women. *Journal of the American Medical Association*.

[11] Alvarez L, Alcaraz M, Pérez-Higueras A (2000). Percutaneous vertebroplasty: functional improvement in patients with osteoporotic compression fractures. *Spine*.

[12] Barr JD, Barr MS, Lemley TJ, McCann RM (2000). Percutaneous vertebroplasty for pain relief and spinal stabilization. *Spine*.

[13] Cyteval C, Sarrabère MP, Roux JO (1999). Acute osteoporotic vertebral collapse: open study on percutaneous injection of acrylic surgical cement in 20 patients. *American Journal of Roentgenology*.

[14] Martin JB, Jean B, Sugiu K (1999). Vertebroplasty: clinical experience and follow-up results. *Bone*.

[15] Gangi A, Dietemann JL, Guth S, Steib JP, Roy C (1999). Computed tomography and fluoroscopy-guided vertebroplasty: results and complications in 187 patients. *Seminars in Interventional Radiology*.

[16] Buchbinder R, Kallmes DF (2010). Vertebroplasty: when randomized placebo-controlled trial results clash with common belief. *Spine*.

[17] Haynes R, Sackett D, Guyatt G, Tugwell P (2006). *Clinical Epidemiology: How to Do Clinical Practice Research*.

[18] Buchbinder R, Osborne RH, Ebeling PR (2009). A randomized trial of vertebroplasty for painful osteoporotic vertebral fractures. *The New England Journal of Medicine*.

[19] Kallmes DF, Comstock BA, Heagerty PJ (2009). A randomized trial of vertebroplasty for osteoporotic spinal fractures. *The New England Journal of Medicine*.

[20] Buchbinder R, Osborne R, Staples M (2009). NEJM correspondence. *The New England Journal of Medicine*.

[21] Kallmes D, Heagerty P, Jarvik J (2009). NEJM correspondence. *The New England Journal of Medicine*.

[22] Mathis JM (2003). Percutaneous vertebroplasty: complication avoidance and technique optimization. *American Journal of Neuroradiology*.

[23] Munk PL, Liu DM, Murphy KP, Baerlocher MO (2009). Effectiveness of vertebroplasty: a recent controversy. *Canadian Association of Radiologists Journal*.

[24] Baerlocher MO, Munk PL, Liu DM (2009). Trials of vertebroplasty for vertebral fractures. *The New England Journal of Medicine*.

[25] Bono CM, Heggeness M, Mick C, Resnick D, Watters WC (2010). North American Spine Society: newly released vertebroplasty randomized controlled trials: a tale of two trials. *Spine*.

[26] Patel U, Skingle S, Campbell GA, Crisp AJ, Boyle IT (1991). Clinical profile of acute vertebral compression fractures in osteoporosis. *British Journal of Rheumatology*.

[27] Silverman SL (1992). The clinical consequences of vertebral compression fracture. *Bone*.

[28] Kallmes DF, Jensen ME (2003). Percutaneous vertebroplasty. *Radiology*.

[29] Klazen C, Lohle P, de Vries J (2010). Vertebroplasty versus conservative treatment in acute osteoporotic vertebral compression fractures (VERTOS II): an open-label randomised trial. *The Lancet*.

[30] Rousing R, Hansen KL, Andersen MO, Jespersen SM, Thomsen K, Lauritsen JM (2010). Twelve-months follow-up in forty-nine patients with acute/semiacute osteoporotic vertebral fractures treated conservatively or with percutaneous vertebroplasty: a clinical randomized study. *Spine*.

[31] Syed MI, Shaikh A (2007). Vertebroplasty: a systematic approach. *Pain Physician*.

[32] O’Brien JP, Sims JT, Evans AJ (2000). Vertebroplasty in patients with severe vertebral compression fractures: a technical report. *American Journal of Neuroradiology*.

[33] Belkoff SM, Mathis JM, Jasper LE, Deramond H (2001). The biomechanics of vertebroplasty: the effect of cement volume on mechanical behavior. *Spine*.

[34] Jensen ME, Evans AJ, Mathis JM, Kallmes DF, Cloft HJ, Dion JE (1997). Percutaneous polymethylmethacrylate vertebroplasty in the treatment of osteoporotic vertebral body compression fractures: technical aspects. *American Journal of Neuroradiology*.

[35] Mathis JM, Barr JD, Belkoff SM, Barr MS, Jensen ME, Deramond H (2001). Percutaneous vertebroplasty: a developing standard of care for vertebral compression fractures. *American Journal of Neuroradiology*.

[36] Chiras J, Depriester C, Weill A, Sola-Martinez MT, Deramond H (1997). Percutaneous vertebral surgery: techniques and indications. *Journal of Neuroradiology*.

[37] Cotten A, Dewatre F, Cortet B (1996). Percutaneous vertebroplasty for osteolytic metastases and myeloma: effects of the percentage of lesion filling and the leakage of methyl methacrylate at clinical follow-up. *Radiology*.

[38] Deramond H, Depriester C, Toussaint P (1996). Vertebroplasty and percutaneous interventional radiology in bone metastases: techniques, indications, contra-indications. *Bulletin du Cancer/Radiothérapie*.

[39] Deramond H, Depriester C, Toussaint P, Galibert P (1997). Percutaneous vertebroplasty. *Seminars in Musculoskeletal Radiology*.

[40] Weill A, Chiras J, Simon JM, Rose M, Sola-Martinez T, Enkaoua E (1996). Spinal metastases: indications for and results of percutaneous injection of acrylic surgical cement. *Radiology*.

[41] Murphy KJ, Deramond H (2000). Percutaneous vertebroplasty in benign and malignant disease. *Neuroimaging Clinics of North America*.

[42] Evans AJ, Jensen ME, Kip KE (2003). Vertebral compression fractures: pain reduction and improvement in functional mobility after percutaneous polymethylmethacrylate vertebroplasty—retrospective report of 245 cases. *Radiology*.

[43] Harrington KD (2001). Major neurological complications following percutaneous vertebroplasty with polymethylmethacrylate: a case report. *Journal of Bone and Joint Surgery*.

[44] Ratliff J, Nguyen T, Heiss J (2001). Root and spinal cord compression from methylmethacrylate vertebroplasty. *Spine*.

[45] Choe DH, Marom EM, Ahrar K, Truong MT, Madewell JE (2004). Pulmonary embolism of polymethyl methacrylate during percutaneous vertebroplasty and kyphoplasty. *American Journal of Roentgenology*.

[46] Padovani B, Kasriel O, Brunner P, Peretti-Viton P (1999). Pulmonary embolism caused by acrylic cement: a rare complication of percutaneous vertebroplasty. *American Journal of Neuroradiology*.

[47] Gaughen JR, Jensen ME, Schweickert PA, Kaufmann TJ, Marx WF, Kallmes DF (2002). Relevance of antecedent venography in percutaneous vertebroplasty for the treatment of osteoporotic compression fractures. *American Journal of Neuroradiology*.

[48] Vasconcelos C, Gailloud P, Beauchamp NJ, Heck DV, Murphy KJ (2002). Is percutaneous vertebroplasty without pretreatment venography safe? Evaluation of 205 consecutives procedures. *American Journal of Neuroradiology*.

[49] Jensen ME, Evans AJ, Mathis JM, Kallmes DF, Cloft HJ, Dion JE (1997). Percutaneous polymethylmethacrylate vertebroplasty in the treatment of osteoporotic vertebral body compression fractures: technical aspects. *American Journal of Neuroradiology*.

[50] Kaufmann TJ, Jensen ME, Ford G, Gill LL, Marx WF, Kallmes DF (2002). Cardiovascular effects of polymethylmethacrylate use in percutaneous vertebroplasty. *American Journal of Neuroradiology*.

[51] Deramond H, Wright NT, Belkoff SM (1999). Temperature elevation caused by bone cement polymerization during vertebroplasty. *Bone*.

[52] Wang GJ, Wilson CS, Hubbard SL (1984). Safety of anterior cement fixation in the cervical spine: in vivo study of dog spine. *Southern Medical Journal*.

[53] Lin EP, Ekholm S, Hiwatashi A, Westesson PL (2004). Vertebroplasty: cement leakage into the disc increases the risk of new fracture of adjacent vertebral body. *American Journal of Neuroradiology*.

[54] Grados F, Depriester C, Cayrolle G, Hardy N, Deramond H, Fardellone P (2000). Long-term observations of vertebral osteoporotic fractures treated by percutaneous vertebroplasty. *Rheumatology*.

[55] Baroud G, Heini P, Nemes J (2003). Biomechanical explanation of adjacent fractures following vertebroplasty. *Radiology*.

[56] Mudano AS, Bian J, Cope JU (2009). Vertebroplasty and kyphoplasty are associated with an increased risk of secondary vertebral compression fractures: a population-based cohort study. *Osteoporosis International*.

[57] Klazen CAH, Venmans A, De Vries J (2010). Percutaneous vertebroplasty is not a risk factor for new osteoporotic compression fractures: results from VERTOS II. *American Journal of Neuroradiology*.

